# Applications of Nanotechnology in the Field of Cardiology

**DOI:** 10.7759/cureus.58059

**Published:** 2024-04-11

**Authors:** Oluwaseyi Oyelaja, Tazkia Najneen, Haroon Alamy, Wendys L Horn, Jose A Niño Medina, Leonor E Duarte, Adila Yaqobi, Palwasha Farooqi, Rohullah Mohammadi, Mohammed Khaleel I. KH. Almadhoun, Bushra Mia Khail, Abed Saeed

**Affiliations:** 1 Medicine and Surgery, New York City Health and Hospitals Corporation (NYCHHC), New York, USA; 2 Paediatrics, Dhaka Medical College and Hospital, Dhaka, BGD; 3 Internal Medicine, Armed Forces Science Academy, Kabul, AFG; 4 Health Sciences, University of Carabobo, Valencia, VEN; 5 Law and Political Sciences, University of Carabobo, Valencia, VEN; 6 Internal Medicine, University of Carabobo, Valencia, VEN; 7 Obstetrics and Gynaecology, Malalai Maternity Hospital, Kabul, AFG; 8 Internal Medicine, Kabul University of Medical Sciences, Kabul, AFG; 9 Medicine and Surgery, Mutah University, Karak, JOR; 10 Internal Medicine, Ali Abad Teaching Hospital, Kabul, AFG; 11 Cardiovascular Medicine, Ali Abad Teaching Hospital, Kabul, AFG

**Keywords:** cad, coronary artery disease, gold nanoparticles, myocardial infarction with no obstructive coronary atherosclerosis, major adverse cardiovascular event, cardiovascular intervention

## Abstract

Cardiovascular diseases (CVDs) are a leading cause of death globally, demanding innovative therapeutic strategies. Nanoformulations, including nanoparticles, address challenges in drug delivery, stem cell therapy, imaging, and gene delivery. Nanoparticles enhance drug solubility, bioavailability, and targeted delivery, with gas microbubbles, liposomal preparations, and paramagnetic nanoparticles showing potential in treating atherosclerosis and reducing systemic side effects. In stem cell therapy, nanoparticles improve cell culture, utilizing three-dimensional nanofiber scaffolds and enhancing cardiomyocyte growth. Gold nanoparticles and poly(lactic-co-glycolic acid) (PLGA)-derived microparticles promote stem cell survival. Stem cell imaging utilizes direct labeling with nanoparticles for magnetic resonance imaging (MRI), while optical tracking employs dye-conjugated nanoparticles. In gene delivery, polymeric nanoparticles like polyethylenimine (PEI) and dendrimers, graphene-based carriers, and chitosan nanoparticles offer alternatives to virus-mediated gene transfer. The potential of magnetic nanoparticles in gene therapy is explored, particularly in hepatocellular carcinoma. Overall, nanoparticles have transformative potential in cardiovascular disease management, with ongoing research poised to enhance clinical outcomes.

## Introduction and background

Cardiovascular diseases (CVDs) are becoming one of the leading causes of death worldwide and are a serious concern. Diabetes mellitus, hypertension, smoking, excessive alcohol use, high cholesterol, and low-density lipoprotein (LDL) levels, truncal obesity, and a sedentary lifestyle with insufficient exercise are common risk factors for CVDs [1]. Many of the medications developed to control and treat CVDs and their consequences have not been able to stop the development or considerably lower the prevalence of these illnesses, despite this fact. Timely reperfusion therapies, such as coronary artery bypass graft (CABG) surgery, thrombolytic therapy, and percutaneous coronary intervention (PCI), are useful for treating myocardial infarction (MI), but they are not appropriate for all patients because of serious complications like bleeding and reperfusion injury [2]. Pharmacological techniques, such as antiplatelet and antiarrhythmic medications, frequently fall short because of side effects, non-targeted drug distribution, and a brief duration of action, which accelerates the development of heart failure. As a result, new treatment approaches have surfaced in the last few years, one such being nanoformulation [3]. A useful therapeutic strategy to lessen the negative effects and non-targeted distribution of pharmaceutical treatment is nanoformulation. Drugs' biological safety, bioavailability, and solubility are improved through nanoformulation, which encapsulates natural ingredients and drug derivatives. Nanomedicines comprise a range of items, such as nanoparticles, nanocomposites, and exosomes [4]. Interestingly, nanoparticles have certain inherent qualities that enable them to play a vital role in advantageous biological processes. These attributes include surface energy, physicochemical characteristics, and surface topographies. Their small size makes it simple for them to pass across cell junctions, opening up a variety of surface modification possibilities. To effectively distribute phytochemicals including curcumin, emodin, gymnemic acid, tilianin, puerarin, berberine, quercetin (QUE), baicalin, naringenin, and others, a variety of organic and inorganic nanoparticles have been produced. Nanoparticles have a variety of functions, including tissue engineering enhancers, circulation-stable nanocarriers, targeted delivery vectors, and behavior regulators for cells [5].

## Review

Nanoparticle vehicles

To address issues like restenosis following percutaneous coronary procedures, a novel method for site-selective administration of therapeutic medicines to parts of the damaged or malfunctioning vascular wall has evolved. This technique, in contrast to stent or device procedures, only requires a single intravenous infusion, allowing for targeted distribution to regions of arterial damage or failure. The technique combines therapeutic drugs with authorized ultrasonic contrast vascular imaging agents, with a specific emphasis on perfluorobutane/dextrose/albumin nanoparticles [6]. Increased adhesion to injured vasculature, non-covalent complexation with specific compounds, and potentiation of substance absorption by cells or tissue are just a few benefits that these nanoparticles have to offer [7].

PGMCs, or gas microbubbles coated with albumin, are an essential part of this method. As a measure of endothelial integrity, these microbubbles show increased adhesion to active or malfunctioning endothelium cells [8]. They can also complex some molecules non-covalently, concentrating them on the particles for possible transmission. When used as ultrasonography contrast agents in different areas, PGMCs have been shown to stick more closely to the vascular wall's depleted or damaged luminal surface [9].

Research conducted in vivo provides evidence for the preferential adhesion of microbubbles to damaged or dysfunctional vasculature. Specifically, the retention of microbubbles has been shown in locations where endothelial dysfunction is caused by variables such as hyperlipidemia [10]. Mechanistically, leukocytes and endothelium both contribute to nanoparticle retention; research has shown that microbubbles cling to the surface of active leukocytes and bind to activated neutrophils [11].

In conclusion, there is strong evidence to support the preferential recruitment and retention of albumin-containing microbubbles, also known as nanoparticles, in areas of compromised or malfunctioning endothelium [12]. With specialized low mechanical index ultrasonic transducers, this device might potentially deliver therapeutic drugs to critical locations for vascular diseases, allowing for the visualization of trapped microbubbles. These results point to a possible effect on lowering vascular events in areas where endothelial dysfunction is present [13].

Nanoparticles in the Treatment of Cardiovascular Diseases

Nanoparticles offer potential applications in treating atherosclerosis by improving overall circulation, enhancing drug solubility, reducing required drug amounts, decreasing drug cytotoxicity, and enabling targeted drug delivery at specific concentrations [14]. Nanoparticle-mediated drug delivery for atherosclerosis and associated complications aims to address inflammation and defective efferocytosis, prevent plaque neovascularization, target macrophages, alter lipid metabolism, prevent neointimal growth, and target thrombosis [15]. For instance, liposomal preparation of bisphosphonate alendronate has shown promise in decreasing neointimal formation and suppressing circulating monocytes in rabbits with iliac artery stenting. Early clinical trials have indicated the safety of liposomal alendronate for infusion during percutaneous coronary intervention [16].

Statins are commonly employed in the treatment of coronary artery disease, but their high-dose administration can result in systemic side effects. Promising alternatives, such as nanocarriers carrying pravastatin and functionalized with oligonucleotides, offer enhanced efficacy and reduced toxicity in nearby tissues [17]. Paramagnetic nanoparticles delivering fumagillin target integrins, leading to decreased systemic adverse effects. Nanocarriers focusing on RNA interference in lipid metabolism, particularly targeting apolipoprotein B and proprotein convertase subtilisin/kexin type 9 (PCSK9), aim to reduce LDL and total cholesterol levels. Lipid-based nanoparticles (lipidoids) targeting PCSK9 in the liver show potential for significant LDL reduction in ongoing clinical trials [17].

Copper sulfide nanoparticles, coupled with antibodies against transient receptor potential vanilloid 1 (TRPV1) ion channels, inhibit lipid accumulation and foam cell formation when administered to apoE-/- mice with atherosclerotic plaques. Nitrogen gas-loaded echogenic liposomes, co-encapsulated with argon, provide controlled delivery to arterial walls, addressing intimal hyperplasia [18]. Nanoparticles exhibit potential in thrombus targeting, anticoagulation, and reperfusion therapy, minimizing bleeding consequences. Theranostic nanoparticles loaded with anti-thrombin attenuate plaque-coagulant activity, improve plaque stability, and repair vascular endothelium [19].

Nanotherapeutic strategies for peripheral artery disease involve nanocarriers enhancing drug retention in plaques and local vascular beds, delivered through catheters or stents. Nanoparticles encapsulating nitrogen gas and RNA interference compounds show promise in inhibiting intimal hyperplasia [20]. Nanodevices, including drug-eluting stents coated with nanoparticles, aim to prevent restenosis and improve endothelial recovery, with sirolimus/pitavastatin nanoparticle-eluting stents showing reduced in-stent restenosis [21].

Polymeric nanoparticles (PLGA) have been utilized to treat plaque destabilization, delivered to plaques through the phagocytosis of monocytes or macrophages. Pitavastatin-coated PLGA nanoparticles reduce monocyte/macrophage infiltration and plaque destabilization. Liposomal siRNA against the CC chemokine receptor 2 (CCR2) inhibits monocyte infiltration, and PLGA nanoparticles loaded with pioglitazone influence macrophage polarity, reducing plaque rupture markers [22-24].

Myocardial infarction, a severe form of ischemic heart disease, has the potential to result in heart failure and mortality. Therapeutic drugs designed to protect the heart aim at enhancing angiogenesis, supporting cardiomyocyte survival, improving heart function, limiting inflammation, and preventing fibrosis [25]. Although virus-mediated gene therapy has shown success in mouse models, safety concerns have restricted its translation to clinical trials. Nanoparticles present a compelling alternative for gene delivery to enhance cardiac function, leveraging their small size, effective penetration, and safer manufacturing methods [25].

Diverse nanoparticle systems, encompassing both entrapping and surface binding methods, serve as carriers for DNA or RNA delivery. PEGylation facilitates gene delivery to vascular tissues, demonstrating heightened transgene expression and diminished choroidal neovascularization in vivo [26]. Mannosylated chitosan nanoparticles exhibit the potential to suppress inflammation and reduce myocardial infarction, with further investigations needed for comprehensive understanding [27].

Placental growth factor (PlGF) emerges as a stimulator of angiogenesis, enhancing cardiac function post-acute myocardial infarction [27]. Chitosan-alginate nanoparticles efficiently deliver PlGF, safeguarding it from enzymatic degradation and amplifying its therapeutic impact at the injury site. Injectable hydrogels, exemplified by RAD16-II loaded with vascular endothelial growth factors, offer a non-invasive drug delivery method, contributing to improved heart function and vascular density in myocardial infarction [28].

Stem cell therapy

Improved Cell Culture

Mitochondrial dysfunction plays a role in cardiac diseases and myocardial infarction. Mitoprotective drugs like cyclosporine-A (CsA) or TRO40303, designed to target cardiac mitochondria, face challenges due to their restricted permeability through plasma and mitochondrial membranes [29]. PLGA nanoparticles carrying mitoprotective drugs, including mdivi-1, demonstrate efficacy in safeguarding cardiomyocytes and reducing myocardial infarct size. Cyclosporine A encapsulated in PLGA nanoparticles proves effective in preventing mitochondrial permeability transition pore opening and mitigating ventricular remodeling in perfusion mouse models [30].

Nanoparticle-mediated therapies exhibit substantial potential for myocardial infarction treatment, encompassing gene delivery and targeted drug administration [31]. These innovative approaches address challenges encountered by traditional treatments, offering promise for enhanced clinical outcomes. Ongoing research and clinical investigations will further deepen our understanding of these impactful nanotherapeutic strategies [32].

Nanoparticles are crucial in advancing cell culture, particularly in overcoming challenges related to stem cell therapy for myocardial infarction (MI). Mimicking the complex structural and chemical conditions of a damaged heart in vitro requires consideration of factors such as the shape of the extracellular matrix, growth hormones, and physical stresses like cell-cell tension and electrical stimulation [33]. Nanotechnology enables the development of 3D nanofiber scaffolds, enhancing nutrient exchange and waste removal with high tensile strength and surface area, ranging from 50 to 500 nm, created through techniques like phase separation, electrospinning, and self-assembly [34].

Studies demonstrate that nanofiber polyglycolic acid and collagen scaffolds significantly enhance cell adhesion compared to larger microfiber-sized scaffolds of the same material. Implementing 3D systems in a bioreactor perfusion setup improves cell attachment and proliferation [35]. Micro- and nanocomposites, in conjunction with a 3D culture system, show promise in promoting stem cell culture. Scaffold shape, as seen in studies using polyethylene glycol (PEG) scaffolds with patterned ridges, influences cell maturation, resulting in improved stem cell retention, growth, and integration [36].

Incorporating gold nanoparticles into scaffolds enhances functionality, leading to the overexpression of crucial molecules like troponin I, alpha-sarcomeric actin, and connexin-43, essential for chemical and electrical communication between cells. Augmenting scaffolds with growth factors such as insulin-like growth factor (IGF-1) and vascular endothelial growth factor (VEGF) further boosts cardiomyocyte growth and function [37]. However, the challenge of prolonged survival of transplanted cells in clinical stem cell therapy persists. Encapsulating cells in microparticles, particularly derived from PLGA and encapsulated with poly-D-lysine and collagen, proves effective in increasing cell survival and retention in targeted tissues [38].

Recent studies highlight that encapsulating cells in microparticles, specifically derived from PLGA and encapsulated with poly-D-lysine and collagen, significantly extends cardiomyocyte retention in mice for up to 2 months. Further improvements involve incorporating specific drug molecules into nanosystems to shield transplanted cells from oxidative stress [39]. For instance, melatonin, an endogenous antioxidant, encapsulated with PLGA-mPEG nanoparticles, demonstrates substantial protection against oxidative injury, both in vitro and in vivo, enhancing stem cell survival during myocardial infarction. These advancements show promise in overcoming challenges in clinical stem cell therapy and improving its effectiveness [40].

Stem Cell Imaging and Tracking

Stem cell imaging and tracking, crucial for monitoring location, survival, and differentiation, utilize direct labeling methods with nanoparticles for their biocompatibility and real-time monitoring advantages. Magnetic nanoparticles like citrate-coated iron oxide and dextran-stabilized superparamagnetic iron oxide nanoparticles (SPIONs) are employed for MRI imaging, especially in liver fibrosis assessment. However, challenges, such as overestimation of survival rates due to macrophage presence, persist [41].

Optical tracking involves dye-conjugated nanoparticles, including gold nanoparticles, silica NPs, carbon nanotubes, and quantum dots, acting as imaging probes. Gold nanoparticles, known for stability and low cytotoxicity, efficiently track stem cells using micro-CT imaging. Other strategies, like upconversion nanoparticles for rat mesenchymal stem cell (MSC) tracking and a reactive oxygen species ROS-responsive self-assembled fluorescent nanoparticle targeting ischemic/reperfused myocardium in a mouse model of MI, promise precise and real-time stem cell imaging across diverse biomedical applications [41].

Nanoparticles in Gene Delivery

Overcoming intracellular and extracellular barriers remains a challenge for nanoparticle-assisted gene delivery. PEI-based nanoparticles, considered gold standard vectors, demonstrate effective transfection, with modifications to reduce toxicity [42].

Dendrimers like polyamidoamine (PAMAM) show promise for gene delivery with enhanced cellular uptake. Graphene-based carriers and chitosan nanoparticles also emerge as novel gene delivery systems. Magnetic nanoparticles hold potential for gene delivery, especially in suicide gene therapy for hepatocellular carcinoma [43]. However, their application in MI is less explored. Advances in these nanoparticle-mediated gene delivery systems provide hope for improving gene therapy efficacy in diverse cardiac applications [44].

## Conclusions

Nanoparticles provide a versatile strategy for combatting global CVDs like MI and atherosclerosis. With unique features facilitating effective drug delivery, targeted imaging, and enhanced cell culture, nanoparticles emerge as valuable tools in cardiovascular medicine. They serve as potent drug carriers, exhibiting promise in treating vascular diseases. Site-selective administration via ultrasound-activated nanoparticles offers targeted drug delivery with potential microbubble visualization. In atherosclerosis, nanoparticles address inflammation, neovascularization, and lipid metabolism through diverse drug delivery strategies. In MI, nanoparticles enable gene delivery, targeted drug administration, and enhanced stem cell therapy, effectively safeguarding cardiomyocytes. Despite existing delivery barriers, innovations in carriers like PEI-based vectors and graphene-based systems hold potential. In summary, nanoparticle-mediated therapies present a multifaceted approach to tackling cardiovascular diseases, with ongoing research promising more effective and targeted treatments in cardiovascular medicine.
